# Refresh my memory: Episodic memory reinstatements intrude on working memory maintenance

**DOI:** 10.3758/s13415-018-00674-z

**Published:** 2018-12-04

**Authors:** Abigail N. Hoskin, Aaron M. Bornstein, Kenneth A. Norman, Jonathan D. Cohen

**Affiliations:** 1 Department of Psychology, Princeton University, Princeton, NJ USA; 2 Neuroscience Institute, Princeton University, Princeton, NJ USA

**Keywords:** Episodic memory, Working memory, Short-term memory, Recollection, Hippocampus

## Abstract

A fundamental question in memory research is how different forms of memory interact. Previous research has shown that people rely on working memory (WM) in short-term recognition tasks; a common view is that episodic memory (EM) only influences performance on these tasks when WM maintenance is disrupted. However, retrieval of memories from EM has been widely observed during brief periods of quiescence, raising the possibility that EM retrievals during maintenance—critically, before a response can be prepared—might affect short-term recognition memory performance even in the absence of distraction. We hypothesized that this influence would be mediated by the lingering presence of reactivated EM content in WM. We obtained support for this hypothesis in three experiments, showing that delay-period EM reactivation introduces incidentally associated information (context) into WM, and that these retrieved associations negatively impact subsequent recognition, leading to substitution errors (Experiment 1) and slowing of accurate responses (Experiment 2). FMRI pattern analysis showed that slowing is mediated by the content of EM reinstatement (Experiment 3). These results expose a previously hidden influence of EM on WM, raising new questions about the adaptive nature of their interaction.

**Electronic supplementary material:**

The online version of this article (10.3758/s13415-018-00674-z) contains supplementary material, which is available to authorized users.

Our memories do not exist in isolation, and neither do the neural circuits that represent them. Experiences may produce transient records in working memory—a temporary store for information to be maintained and manipulated over delays of seconds (Baddeley, 1992; Baddeley & Hitch, 1974; Repov & Baddeley, 2006). Experiences can also simultaneously lay down more lasting traces as episodic memories, available to be recalled at a later time (beyond minutes), allowing us to relive specific, previously experienced events tied to the time and place of their occurrence (Tulving, 1983).

Early models proposed that working memory and long-term memory operated wholly in parallel (Shallice & Warrington, 1970). Evidence for the dissociation between working memory and episodic memory largely came from lesion studies, which found that damage to the medial temporal lobe (MTL) caused severe episodic memory deficits (Cave & Squire, 1992; Squire, 1992), while working memory, associated with the prefrontal cortex (Cohen et al., 1994), remained intact (Drachman & Arbit, 1966). More recent models propose that they support each other (Baddeley & Hitch, 2000; Cohen & O’Reilly, 1996). There is accumulating evidence that episodic memory, and its neural substrates in the MTL, are engaged during short-term memory tasks that also engage working memory (Axmacher et al., 2007; Lewis-Peacock, Cohen, & Norman, 2016; Ranganath, 2005; Ranganath & Blumenfeld 2005; Ranganath, Cohen, Dam, & D’Esposito, 2004; Ranganath, D’Esposito, Friederici, & Ungerleider, 2005), suggesting these memory systems do not operate entirely independently of one another.

Experiments testing for an interaction between episodic memory (EM) and working memory (WM) have historically focused on the hypothesis that EM is used to support WM when maintenance is disrupted, leading to errors that reflect features of EM. For instance, participants show proactive interference from recently studied stimuli when WM is disrupted for 18 seconds (Wickens, Dalezman, & Eggemeier, 1976). However, subsequent research suggests that EM may contribute to WM more ubiquitously, even when WM is not disrupted during 4-second delays (Atkins & Reuter-Lorenz, 2008, 2011). Here, we investigate the nature of these interactions to ask *how* EM contributes to undisrupted WM.

## Do ongoing reinstatements from episodic memory influence working memory, even in the absence of distraction?

A growing number of studies indicate that during periods of rest, the neural structures that support EM are active (Buckner, 2010) and appear to be reinstating recent experiences (Tambini, Ketz, & Davachi, 2010) or activating potential future scenarios constructed on the basis of past experiences (Buckner & Carroll, 2007). These reinstatements trigger coordinated activity patterns across a broad swath of cortical regions, including those presumably involved in WM maintenance, such as the prefrontal cortex (Miller & Cohen, 2001). This widespread activation is reliably present even during brief lapses in external stimulation (Logothetis et al., 2012), such as those typically used as maintenance periods in WM experiments.

These observations lead us to ask the question: How do ongoing reinstatements from EM affect the content of WM, even when the latter is not being disrupted? We hypothesized that an influence of EM on WM search might be observable by more sensitive measures than substitution errors during recall: through examination of reaction times (Atkins & Reuter-Lorenz, 2008, 2011) and the use of content-specific pattern analysis in neuroimaging.

## Using context as a signature of episodic memory

To test our hypothesis, we leverage the fact that retrievals from EM carry with them temporal and associative context (Howard & Kahana, 2002), such that triggering the recall of one memory from a given context can cause the subsequent, involuntary recall of other memories sharing that context (Bornstein & Norman, 2017; Hupbach, Gomez, & Nadel, 2009). This can occur even at the short delays typically associated with WM (Hannula, Tranel, & Cohen, 2006). Therefore, we reasoned that if reinstatements from EM occurred during WM maintenance, then these reinstatements would likely be of memories that shared an encoding context with the target stimuli. Even if these reinstated memories do not lead to overt errors, they may intrude on or degrade other, task-relevant representations being maintained in WM, and thereby affect search and response times on subsequent decisions—even several seconds later, and even in the absence of further EM reinstatement (Atkins & Reuter-Lorenz, 2008). They may also express themselves in patterns of neural activity reflective of the reinstated memories.

It is also possible that episodic memories are reinstated at the moment of retrieval instead of or in addition to during WM maintenance. Research on prospective memory, a memory task in which an individual must remember to perform an action at a target event in the future (e.g., remembering to stop at the supermarket on the way home; see Brandimonte, Einstein, & McDaniel, 1996), point to a reason why EM reinstatements only at probe could be strategic. Constantly monitoring the environment for the target event is cognitively costly; relying on environmental context clues to reinstate the intended action at the relevant decision point (e.g., getting into the car after work) could free cognitive resources for other tasks during the delay (McDaniel & Einstein, 2000). Measuring the timing of memory reinstatements using neuroimaging over the course of a task can help address whether EM context reinstatements are ongoing or locked to retrieval.

## Present study: Three experiments measuring how episodic memory reinstatements can inject contextual associates into working memory, even in the absence of distraction

We present three experiments testing the hypothesis that context reinstated from EM intrudes on WM maintenance. In Experiment 1, we show that participants substitute same-context items in response to interference in a classic short-term delayed-recall task with distraction during the maintenance period. These intrusions are distinct from the recency effect traditionally used to identify episodic influence in this task. In Experiment 2, we show that the influence of reinstated context is evident in response times, even when accuracy is at ceiling. In Experiment 3, we repeat the task from Experiment 2 with fMRI, and use multivariate pattern analysis (MVPA) to generate a trial-by-trial neural measure of how likely it was that participants were recalling a specific past context. We use this neural index of reinstatement to predict the degree of response-time bias on a given trial. Finally, we show that EM reinstatement affects responses via a specific effect on the contents of WM during the maintenance period.

Together, the results of these experiments reveal a novel aspect of the interaction between EM and WM: When target items are stored in WM, ongoing reinstatements from EM can inject contextual associates of these targets into WM, leading to confusion about whether these associates were part of the target set.

## Experiment 1

**Fig. 1:**
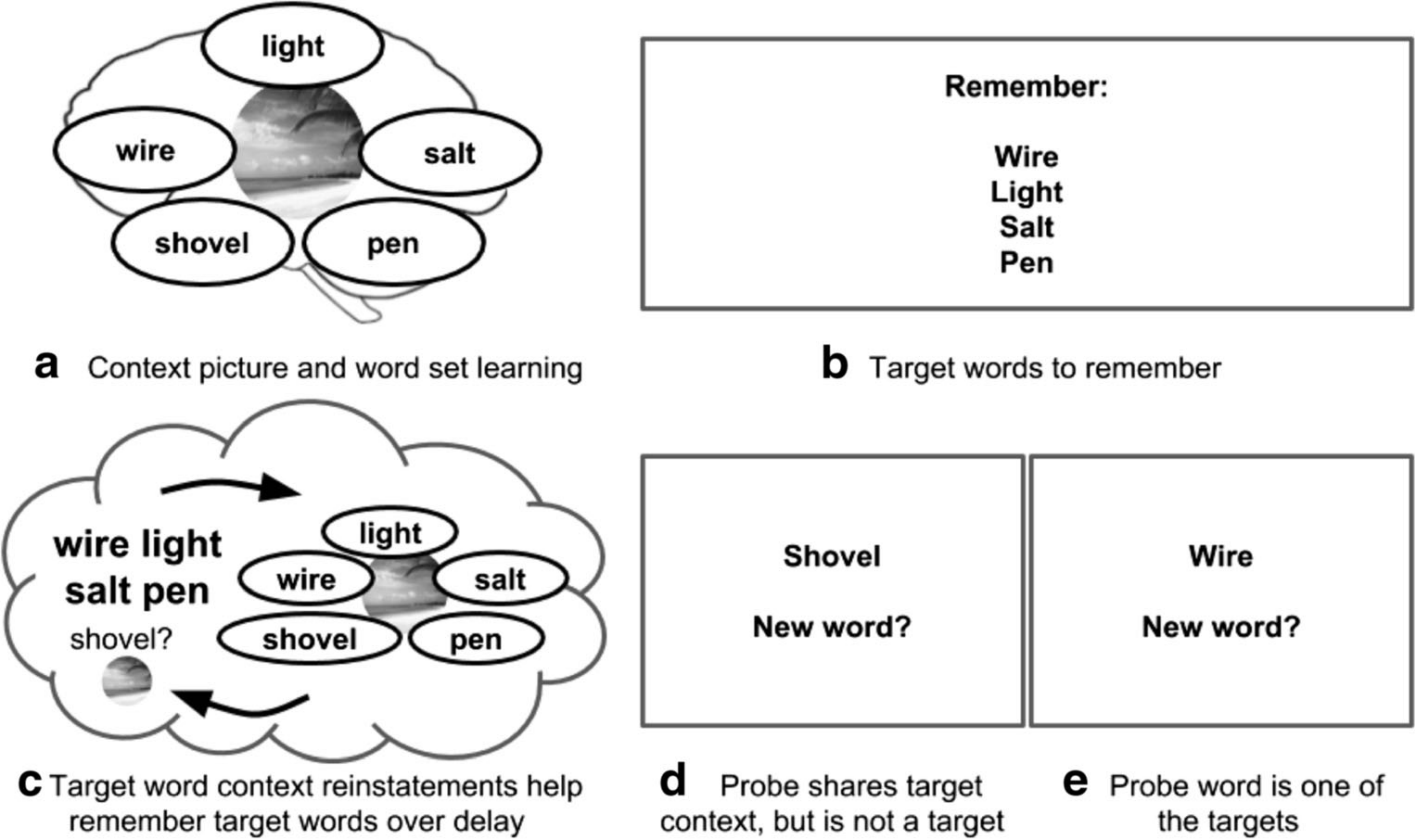
Episodic memory can inject incidental information into working memory. **a** Episodic memory encodes items along with the context in which they were learned. **b** When presented with target items to maintain over a delay period, working memory maintenance may be periodically influenced by reinstatements from episodic memory. **c** These reinstatements may contain other items sharing the encoding context of the target items. **d** These items might affect subsequent behavior, by impeding decision-making when these items support the incorrect decision, **e** and/or by facilitating decision-making when they support the correct decision

**Fig. 2:**
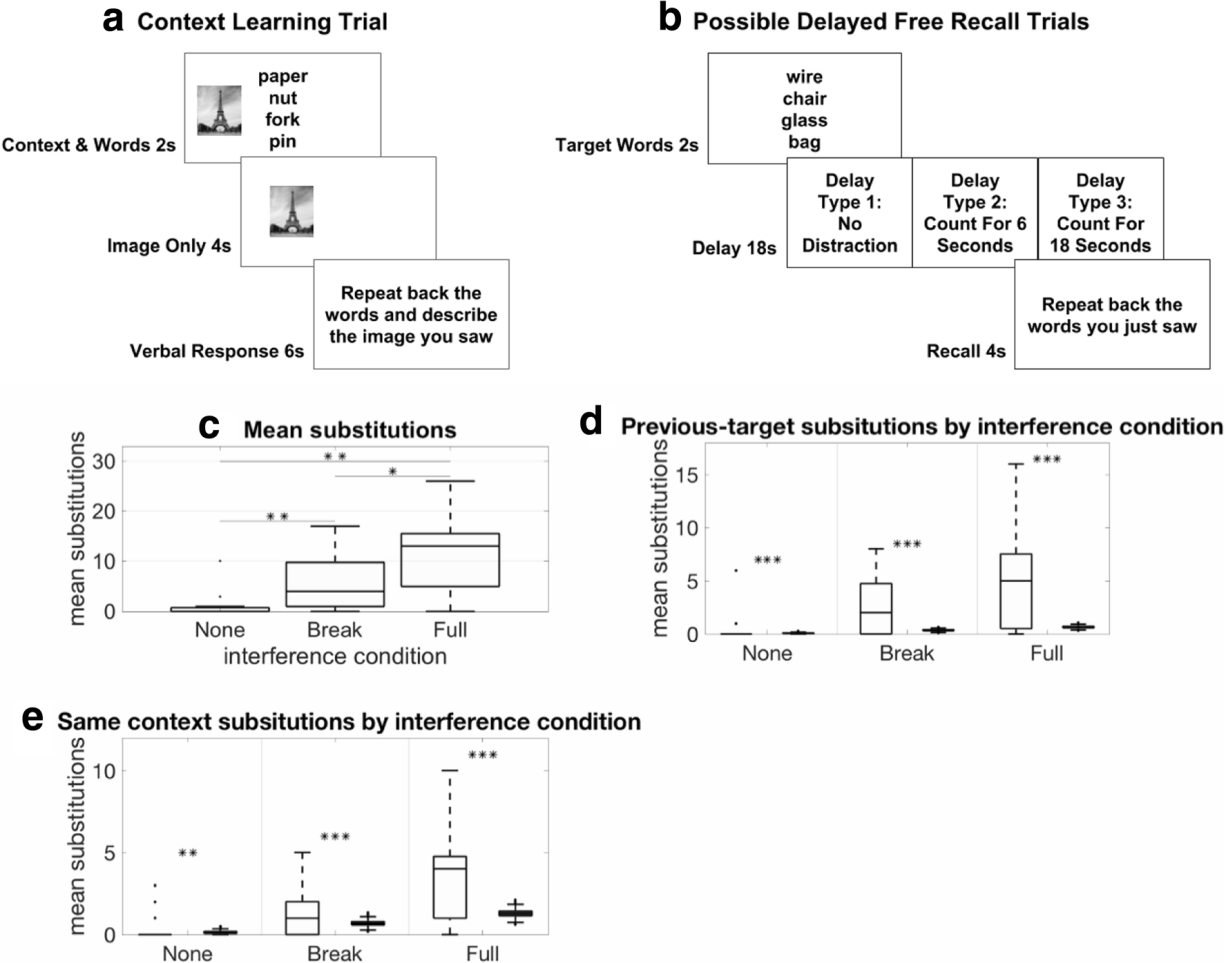
Experiment 1: Free-recall task with added context. **a** Participants (*n* = 15) studied lists of words in contexts distinguished by different pictures. **b** We probed how these contexts affect performance on a short-term recall task under three conditions: (1) when working memory was not disrupted, (2) briefly disrupted (break distraction), or (3) completely disrupted (full distraction). **c** Participants made more errors in the distraction conditions compared to the no distraction condition (*p* < .01 for all comparisons,  paired, two-sided *t* tests). *p < .05, **p < .01, ***p < .001. Black horizontal lines within boxes indicate median substitutions. Bottom and top edges of the box indicate the 25th and 75th percentiles. Whiskers extend to the most extreme data points not considered outliers. Black points outside boxes indicate outliers. **d** Within each interference condition, left bars reflect subject data and right bars reflect simulated data based on randomized substitutions from the experiment’s word set. In all three conditions, participants made errors that reflected the influence of reinstated context. Specifically, participants substituted words from the previous trial at a higher rate than would be expected if they were randomly substituting words previously learned in the experiment. As computed by bootstrap analysis, the number of previous trial substitutions was greater than chance on full-interference (*p* < .001), break-interference (*p* < .001), and no-interference trials (*p* < .001). **e** Participants also made substitution errors during recall that reflected the encoding context of the target set, or *same-context* errors, at a higher rate than would be expected if they were randomly substituting words previously learned in the experiment. As computed by bootstrap analysis, the amount of *same-context* errors made was greater than chance on full-interference (*p* = .001), break-interference (*p* = .001), and no-interference trials (*p* = .025). Box plots follow the same conventions as in **d**

Previous studies using short-term recall tests have found that distraction during delay periods causes participants to rely on EM rather than WM, as evidenced by the fact that errors are primarily words substituted from recent trials (Brown, 1958; Lewis-Peacock et al., 2016; Peterson & Peterson, 1959; Rose, Buchsbaum, & Craik, 2014; Zanto, Clapp, Rubens, Karlsson, & Gazzaley, 2016). Here, we tested whether these substitutions can be biased by the encoding context of the target words. Specifically, if the four target words are sampled from one of the 12-word encoding contexts established at the outset of the experiment, does this lead to substitution of other (nontarget) words from the same context? The logic of the study is shown in Fig. 1, and examples of the initial context learning and delayed recall trials are shown in Fig. 2a–b.

### Methods and materials

#### Participants

Fifteen Princeton psychology students (nine females; ages 18–22 years) completed the study for course credit. All participants had normal or corrected-to-normal vision and provided informed consent. The study protocol was approved by the Princeton University Institutional Review Board.

#### Stimuli

The experiment used six scene pictures, each of which served as a “context” that uniquely linked one of six sets of 12 words. The words and context pictures were not organized by semantic category; instead, the words used in each set and the image associated with each set were randomized across participants. The pictures were color photographs of famous outdoor landmarks. The words were concrete nouns drawn from the Medical Research Council Psycholinguistic Database (Wilson, 1988). All words had a maximum of two syllables, Kucera–Francis written frequency of at least 2, a familiarity rating of at least 200, a concreteness rating of at least 500, and an imageability rating of at least 500.

#### Procedure

##### Word-context learning trials

The goal of the initial context learning phase was to associate words with distinct encoding contexts. On each of 48 learning trials, participants were shown four words drawn from the same set alongside the photograph associated with that set (see Fig. 2a). The picture served as an encoding context. To help participants encode the 12 words associated with the same picture as all belonging to the same context, each word was presented three times along with three other words randomly sampled from the same set and always displayed in the same context (i.e., with the picture associated with that list). On each trial, the four words and the picture associated with those words were presented for 2 seconds before the words disappeared and the picture remained on-screen. Four seconds later, the context picture was replaced by a prompt asking participants to vocally repeat back the four words just shown, and to then briefly describe the picture they had just seen. Participants were given 6 seconds to respond. Trials were of fixed length, regardless of participant’s responses.

##### Free-recall phase

After the learning block was completed, participants performed 54 trials of a short-term retention task. On each trial, participants were shown four target words. The four target words were all drawn from the same context. No picture was presented alongside the words. Words remained on the screen for 2 seconds and were followed by an 18-second delay.

There were three types of delay (see Fig. 2b). Delay trial types were randomly intermixed, with 18 trials of each type. In the no-distraction condition*,* participants were shown a fixation cross, in the center of the screen, for the entirety of the 18-second delay. In the break-distraction condition, participants were shown a fixation cross in the center of the screen for 6 seconds. After 6 seconds, participants were shown a randomly generated three-digit number in the center of the screen. The number served as a prompt to count down out loud by sevens, starting at that number. After 6 seconds of counting, participants were again shown a centered fixation cross for 6 more seconds. In the full-distraction condition, participants were shown a three-digit number at the start of the delay period, and instructed to count backwards out loud by sevens, starting from the prompted number, for the entire delay period.

In all conditions, participants were given 8 seconds after the delay period to vocally recall the words shown at the beginning of the trial. These responses were recorded and scored for the number of words correctly recalled (zero through four). Mistakes were categorized as one of three types: (1) words from the same encoding context as the targets, (2) words from the previous free recall trial, or (3) other words learned during the experiment but not in Categories 1 or 2. (No substitutions were made using words that were not learned during the experiment.)

### Experiment 1 results

We expected to see increasing numbers of substitution errors as the demands on working memory increased; therefore, we predicted participants would make the fewest substitutions following delays with no distraction, and the most substitutions following full distraction.

Consistent with our predictions, participants made more errors in the full-distraction condition than in the break-distraction condition, *t*(14) = 3.2756, *p* < .01, paired, two-sided *t* test, and the no-distraction condition, *t*(14) = 6.4526, *p* < .001, and more errors in the break-distraction condition than in the no-distraction condition, *t*(14) = 4.4852, *p* < .001 (see Fig. 2c).

We also predicted that distraction would increase reliance on episodic memory and, accordingly, that substitution errors would reflect information retrieved from episodic memory. To test this hypothesis, we marked errors as belonging to one of three categories—two that specifically reflected intrusions from episodic memory: *previous-target* substitutions and *same-context* substitutions, as well as *other errors,* which reflected intrusions or failures of other kinds. These categories were motivated by the following considerations. First we expected recently experienced words—in particular, the four words from the trial immediately previous—to be most accessible in episodic memory and therefore likely to be recalled, brought into working memory, and mistakenly invoke a target response. We refer to these as *previous-target* substitutions. Second, we expected that maintaining target words in working memory would trigger episodic memory reinstatement of the context in which these words were studied (Gershman, Schapiro, & Hupbach, 2013; Howard & Kahana, 2002). If this occurs, we should see an elevated substitution rate for the eight words that were studied in the same context as the target words, but that were not part of the current trial’s target set. We refer to these as *same*-*context* substitutions. The context from which the target words were drawn changed with each trial, ensuring that *previous-target* and *same-context* substitutions were mutually exclusive possibilities. Finally, we refer to substitutions from one of the 56 remaining words learned in the experiment, that were neither targets, *previous-target* or *same-context* errors, as *other* errors.

By categorizing errors in this way, we could compare the number of each kind of error to the number that would be expected if the errors were drawn at random from the 68 possible nontarget words. While all three kinds of words should be present in episodic memory, we predicted that *previous-target* errors, reflecting recency, and *same-context* errors, reflecting the bias toward clustered recall of items sharing encoding context, should be overrepresented relative to *other* errors.

If substitution errors were uniformly distributed among the 68 possible words, only 4/68 of the errors made in each interference condition should be *previous-target* substitutions. Participants substituted words from the previous trial at a higher rate than would be expected if they were randomly substituting words previously learned in the experiment (see Fig. 2d). As computed by bootstrap analysis, the amount of previous trial substitutions made was greater than chance on full interference (subject mean = 5.20, *SD* = 4.95; bootstrapped mean = .64, *SD* = .10; *p* < .001), break interference (subject mean = 2.67, *SD* = 3.04; bootstrapped mean = .34, *SD* = .08; *p* < .001), and no interference trials (subject mean = .47, *SD* = 1.55; bootstrapped mean = .07, *SD* = .04; *p* < .001). This suggests that information from previous trials from episodic memory entered working memory, even when working memory was not overloaded.

Similarly, if substitution errors were uniformly distributed among the 68 possible words, only 8/68 of the errors made in each interference condition should be *same-context* substitutions. Instead, on full interference trials, the proportion of *same-context* substitutions was greater than what would be expected by chance (subject mean = 3.40, *SD* = 2.77; bootstrapped mean 1.29, *SD* = .21; *p* = .001). This suggests that context information was indeed affecting decision-making when working memory was overloaded (see Fig. 2e). *Same-context* substitutions were also greater than what would be expected by chance in the break condition (subject mean = 1.33, *SD* = 1.59; bootstrapped mean = .86, *SD* = .19; *p* = .001). Critically, although the frequency of substitutions on the no-interference trials was low (mean = 1.13, *SD* = 2.67; see Fig. 2c), when they did occur, they were biased toward coming from the same context as the target words (subject mean = .40, *SD* = .91; bootstrapped mean = .13, *SD* = .08; *p* = .025).

### Experiment 1 discussion

Participants completed a short-term retention task with three distraction conditions. When there was no distraction during the retention delay, participants made almost no errors, consistent with the idea that they were able to easily use working memory to complete this task. Errors increased when participants were made to perform a distractor task midway through the delay, and were further increased when the distractor task spanned the entire retention interval. These errors took the form of substituting other words from the experiment in place of the current trial’s target words.

A disproportionate number of substitutions were made using words from the same encoding context as the target words, despite the fact that these kinds of words represented only a small fraction of the words used on the task. This distribution of substitutions is consistent with previous observations that, when working memory maintenance is interrupted, participants rely on recency-biased retrievals from episodic memory (Lewis-Peacock et al., 2016; Rose et al., 2014; Zanto et al., 2016). Critically, our results also establish that the context-based nature of errors can serve as an additional signature of episodic memory recruitment in these tasks, augmenting the suite of tools available to identify EM recruitment. As would normally be predicted, both kinds of errors were most evident when retention in working memory was subject to interference. Notably, however, the pattern of errors indicated the engagement of episodic memory even when distraction was momentary, hinting that it might be present even in the absence of distraction—that is, under conditions ordinarily assumed to rely exclusively on working memory.

Our findings raise two questions: First, does episodic memory affect working memory in the absence of external distraction? While substitutions in the no-distraction condition were significantly biased toward being from the same encoding context as the target words, there were very few errors (of any kind) in this condition, making us wary of drawing strong conclusions from this result on its own. Second, when during the task does episodic-memory retrieval occur, and how does it influence performance? Are episodic memories retrieved during the delay, either incidentally and/or to support maintenance, or strictly at the time of response? We use the signature of context effects established in Experiment 1 to address these questions in Experiments 2 and 3.

## Experiment 2

**Fig. 3:**
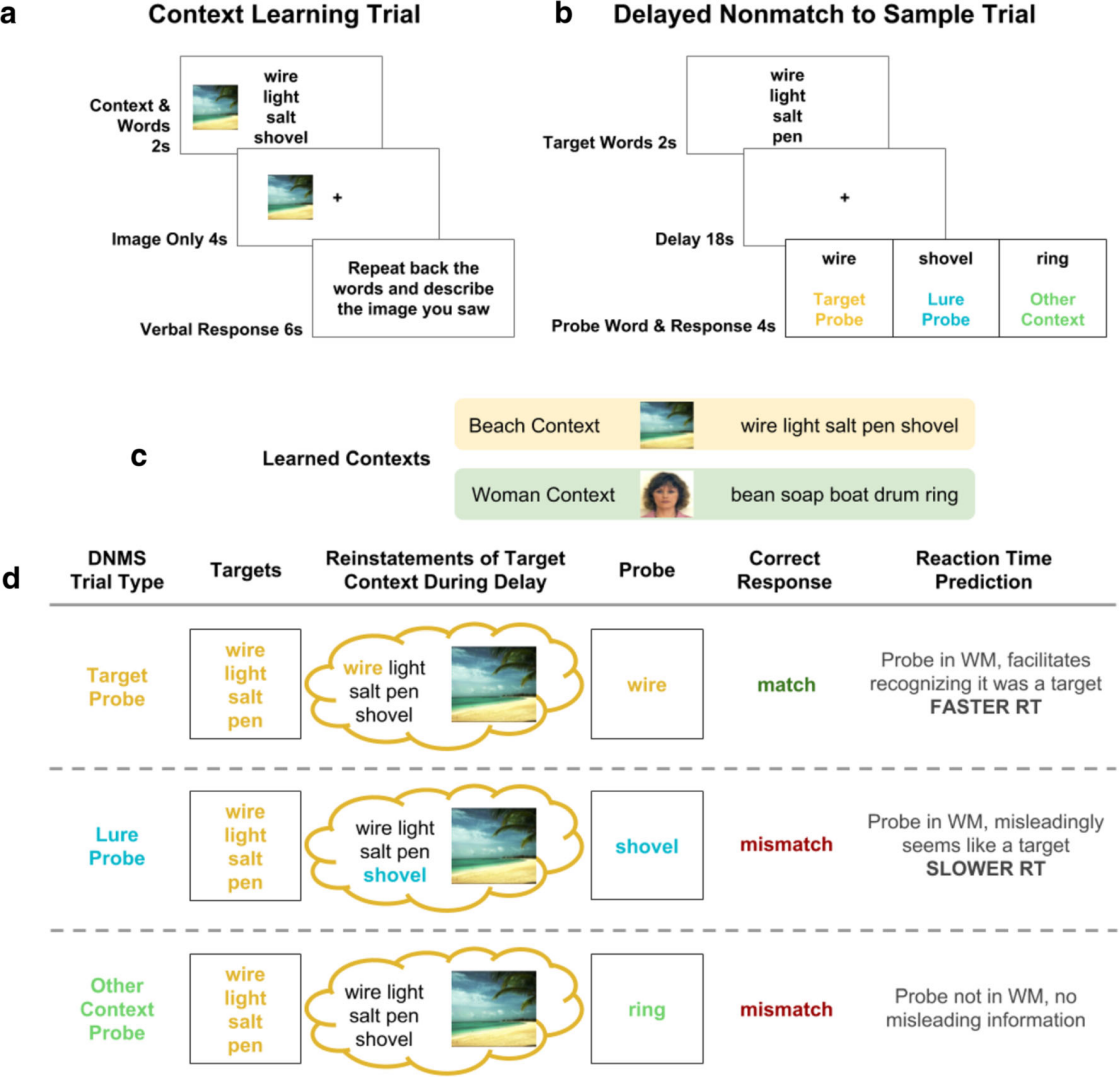
Experiment 2: DNMS task with added context. **a** In the context-learning phase, participants studied four sets of words, each set paired with a unique context picture.**b** In the testing phase, participants performed a delayed-nonmatch-to-sample (DNMS) task, in which they remembered four target words across an 18-s delay. After the delay, they were shown a single probe word and asked whether that word was *not* one of the four they had just seen. Response times were recorded and used as a measure of whether the participants’ performance had been affected by context information reinstated from episodic memory. **c** Subsets of two example contexts are presented for illustrative purposes. **d** We hypothesized that the contents of working memory are influenced by reinstatements from episodic memory. These reinstatements activate working-memory representations of trial-irrelevant words that were linked to the target words during the context-learning phase. We predicted that, when the probe word was one of the targets, participants would be fastest to respond because the target probe should clearly match the content of working memory, allowing the search process to terminate quickly. For nontarget probe trials, we predicted participants would respond more slowly because they needed to exhaustively search through the contents of working memory to decide to reject the probe. Within nontarget probe trials, we predicted participants would be slowest to respond to lure probes, because these probes would match the context information in working memory elicited by the target words but mismatch the actual target words. Because this conflicting evidence was not present in other-context probe trials—the probe word did not match the context information or target words in working memory—we predicted participants would be less impaired on other-context probe trials

In Experiment 2, we used a more sensitive measure, reaction time (RT), to investigate the effect of context on behavior. Participants performed the same context training exercise from Experiment 1 (see Fig. 3a), this time followed by a delayed-nonmatch-to-sample task (DNMS; Fig. 3b), with no distractions during the delay periods.

### Methods and materials

#### Participants

Eighty-eight Princeton students (55 females; ages 18–21 years; native English speakers) completed the study for course credit. All participants had normal or corrected-to-normal vision and provided informed consent. The Princeton University Institutional Review Board approved the study protocol. Eight participants were excluded from RT analyses on the basis of their accuracy scores being less than chance performance, leaving the participants reported here.

#### Procedure

In the learning phase, participants studied four different sets of words, each containing 12 words drawn from the same set of words used in Experiment 1. Each word set was paired with a unique context picture. The paired words and orientation of each context picture were randomly assigned anew for each participant. Learning-phase trials followed the same procedure as in Experiment 1 (see Figs. 2a and 3a), now over four contexts of 12 words each.

In the testing phase, participants performed 60 trials of a DNMS task, in which targets were selected from the words learned in the learning phase (see Fig. 3b). On each trial, one context was selected at random, and then four target words were selected from within that context. These words were shown on the screen together for 2 seconds—critically, without the associated context image. When the words disappeared, they were replaced by a centered fixation cross, displayed for 18 seconds. Participants were instructed to use this delay to remember the four words they had just seen. There was no distraction during the delay period.

After the delay period, participants were shown a probe word and asked to respond “mismatch” if the given word was not one of the four they had just seen on this trial, or “match” if it was one of the four target words. The keys used to signify *mismatch* and *match*—the left and right arrows—were counterbalanced across participants. A successful response was indicated by a green fixation cross, while an unsuccessful response (incorrect response or time-out after 4 seconds) was indicated with a red fixation cross.

Probe words could be one of three types: (1) target probes were drawn from the four-word target set presented on the current trial; (2) lure probes were drawn from the same context list as the target words, but, critically, these probes were not one of the target words; (3) other-context words were drawn from one of the three contexts other than the one from which the target words were drawn. Target probes were drawn from the target words, so the correct response to target probes was that they were a “match” to the targets; lure and other-context probe words did not contain one of the target words, so the correct response on lure and other-context probe trials was “mismatch.” Participants were not signaled as to which kind of probe was being used on each trial.

There were equal numbers of target, lure, and other-context probe trials, so a participant who responded “mismatch” on every trial would be correct on 66% of trials. Eight participants fell below this accuracy threshold, whom we excluded from further analysis.

### Experiment 2 results

#### Accuracy

**Fig. 4:**
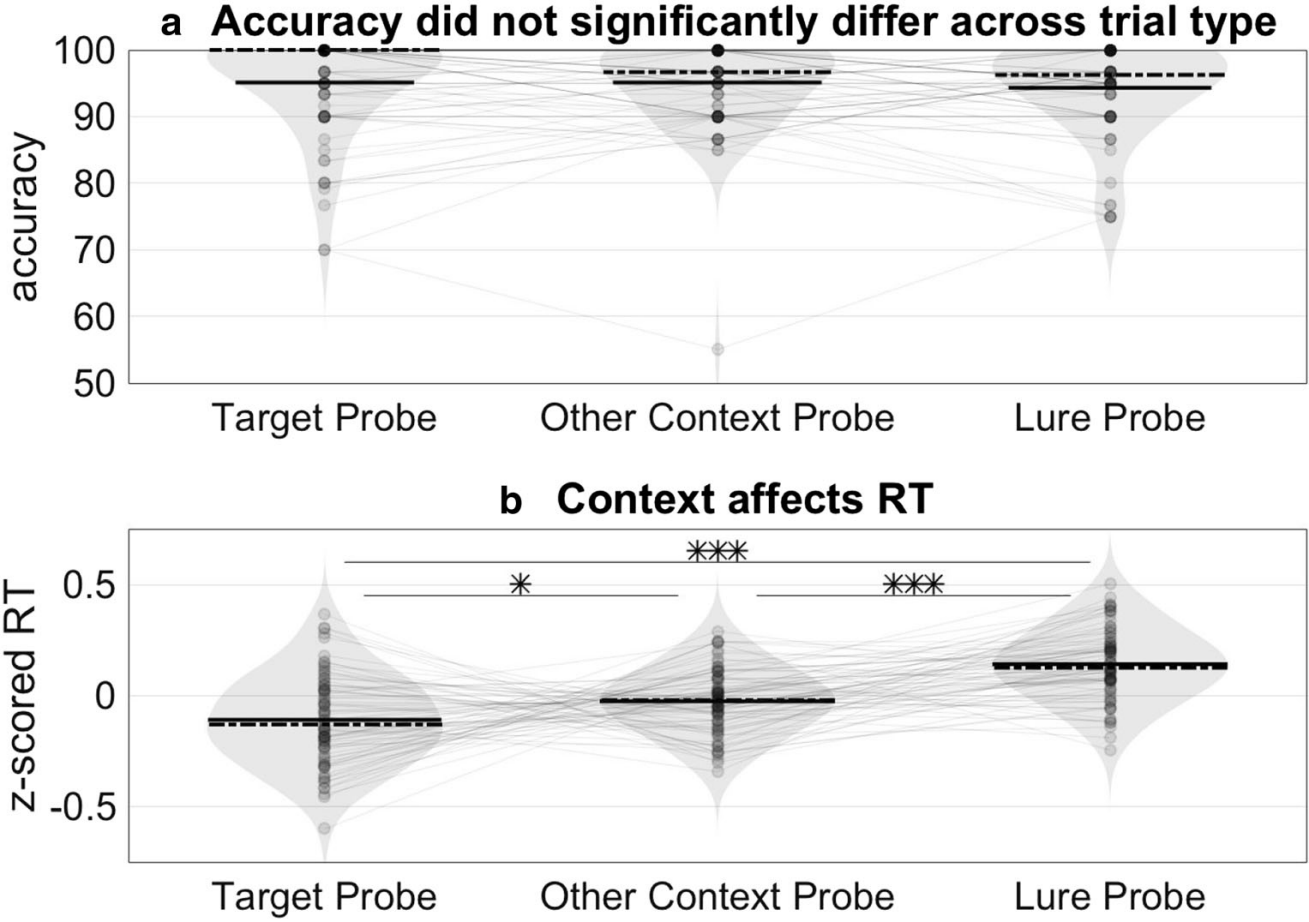
Study 2 results: Response times reflect influence of study context. **a** For participants with above-chance performance (*n* = 80), accuracy was high across all three conditions (mean = 94.84%, *SEM* = .78%) with no difference in accuracy between target (mean = 95.01%, *SD* = 7.37%), other-context (mean = 95.10%, *SD* = 6.59%), or lure trials (mean = 94.31%, *SD* = 6.94%, *p* > .2 by paired, two-sided *t* tests for all pairwise comparisons). Solid lines reflect mean accuracy. Dashed lines reflect median accuracy. **b** RTs were log-transformed and *z*-scored within subject to control for individual differences in mean RTs and nonnormal RT distributions. Task-irrelevant context information slowed RTs; using paired, two-sided *t* tests, we found that participants responded slower to *lure probes* (mean *z*RT = .14, *SD* = .16) than to *target probes* (mean zRT = −0.11, *SD* = .20), *t*(79) = −6.7603, *p* < .001, or *other-context* probes (mean zRT = −0.03, *SD* = .14), *t*(79) = −6.8583, *p* < .001. *p < .05, ***p < .001

Given the absence of distraction, accuracy was high across all three conditions (mean = 94.84%, *SEM* = .78%) with no significant differences in accuracy between target (mean = 95.01%, *SEM* = .82%, other-context (mean = 95.10%, *SEM* = .74%), or lure trials (mean = 94.31%, *SEM* = .78%, *p* > .2 by paired, two-sided *t* tests for all pairwise comparisons; see Fig. 4a). Because these inaccurate trials were rare and did not vary in proportion between categories, we excluded inaccurate trials from the RT analyses.

#### Reaction times

We predicted that participants would on average respond fastest to target probes, as the probe word would most reliably match the contents of working memory (see Fig. 3d). In contrast, nontarget probe trials, in which the probe word did not match any of the targets, would be slower because they required an exhaustive search of the contents of working memory to decide on rejection (a prediction that follows from both serial and parallel models of working memory search; Sternberg, 1969; Ratcliff, 1978).

On nontarget probe trials, which included lure and other-context probes, participants had to make the same response: to reject the probe word as one of the targets. Thus, any difference in RT between these two trial types could not be attributed to differences in the required response.

Within nontarget probe trials, we predicted that participants would be slower to respond to lure than to other-context probes: If context reinstatement from episodic memory activates trial-irrelevant words from the same context as the target words, then lure words can become activated in working memory. If this occurs, activated lure information will match lure probes, increasing uncertainty and slowing “mismatch” responses to these probes. Other-context probes would not induce such uncertainty, since they would neither match the targets nor would they match reinstated lure information.

Response times were log-transformed and *z*-scored within subject to control for individual differences in mean RTs or nonnormal RT distributions; however, the results reported below are also present in the raw RTs (see Supplemental Fig. S1).

Using paired, two-sided *t* tests, we found that participants responded fastest to target probes (mean zRT = −0.11, *SEM* = .02) compared with lure probes (mean zRT = .14, S*EM* = .02), *t*(79) = −6.7603, *p* < .0019 (see Fig. 4b), or other-context probes (mean zRT = −0.03, *SEM* = .02), *t*(79) = −2.4133, *p* = .018. Critically, we found participants responded slower to lure probes than to other-context probes, *t*(79) = −6.8583, *p* < .001 (see Fig. 4b). The latter is noteworthy as the only difference between lure and other-context probes is whether the probe word was learned in the same context as the target during the task-irrelevant part of the experiment.

### Experiment 2 discussion

In Experiment 2, participants performed a DNMS task using study words that had previously been associated with one of four separate contexts. The lack of distraction and the relatively short (18 second) delay period were chosen to make it easy for participants to rely solely on working memory to perform the task. Indeed, as has been repeatedly observed in tasks with this kind of structure, accuracy was near ceiling and did not differ across trial types. However, we observed an effect of encoding context on response times. Specifically, while responses to target probes were faster than responses to both kinds of nontarget probes, responses to lure probes—those sharing an encoding context with the target—were slower than responses to probes from any of the other three contexts.

This result is particularly striking because it is in the opposite direction of what would be expected if responses were simply biased toward the more prevalent response type (mismatch). If this were the case, then participants should be faster to respond to lure or other-context probes (two thirds of trials), rather than target probes (one third of trials). Instead, the results support the idea that responses may reflect deliberative accumulation of information from working memory, and that this process can be slowed by the intrusion of countervailing information: the context-driven reinstatement of lure words from episodic memory. These reinstatements need not catastrophically interfere with maintenance—rather than occupying discrete “slots” in working memory, they may simply reduce the fidelity of the representation of the target set (e.g., Ma, Husain, & Bays, 2014), slowing the integration process without producing an incorrect response.

Note that the same logic should apply irrespective of whether the probe is a lure or an other-context probe—if the correct response is “mismatch,” but (during the delay) participants mentally reinstate the context matching the probe, then this should lead to slower RTs to that probe. However, reinstatements of the target-word context should be much more frequent than reinstatements of other contexts, which would explain why responses to lure probes (from the target context) are slower, on average, than are responses to other-context probes.

## Experiment 3

Experiment 2 demonstrated that encoding context has an effect on responses following a delay, even in the absence of distraction. We interpret this result as following from putative episodic memory reinstatements during the delay period. We reasoned that this effect, observed in Experiment 2 as an average across trials, should be determined on a trial-by-trial basis by whether episodic memory reinstatement of the probe context occurred on that trial, as well as which memories were reinstated. To directly test this, in Experiment 3, we had participants perform the same distraction-free DNMS task from Experiment 2 while being scanned using functional magnetic resonance imaging (fMRI), which allowed us to use multivariate pattern analysis (MVPA) to measure the content of memory reinstatement on each trial.

### Methods and materials

#### Participants

Forty healthy participants (26 females; ages 18–30 years) were recruited. All participants had normal or corrected-to-normal vision and provided informed consent. The Princeton University Institutional Review Board approved the study protocol. Exclusion criteria for recruitment included the presence of metal in the body, claustrophobia, neurological diseases or disorders, tattoos above the waist, pregnancy, not speaking English as a native language, and left-handedness. Four participants were excluded from the final analyses for the following reasons: excessive movement in the scanner—defined as maximal instantaneous displacement larger than 3 mm across any individual scanner run (two participants), or numerically below-chance accuracy on the DNMS task (two participants). Data are reported for the remaining 36 participants.

#### Stimuli

The fixation training phase used scene and scrambled scene pictures that were not used in any other phase of the experiment. In the context learning phase*,* participants learned four word sets each with its own context picture. The pictures were either faces or scenes. The face pictures were emotionally neutral and of nonfamous individuals, taken from the Psychological Image Collection at Stirling University (PICS; http://pics.stir.ac.uk). The scene pictures depicted two natural, nonfamous places. One of the faces and one of the scenes were always displayed on the left side of the screen; the other face and other scene were always displayed on the right side of the screen. Thus, each set was associated with one of the following context stimuli: a face on the left, a face on the right, a scene on the left, or a scene on the right. The test phase followed the same DNMS procedure used in Experiment 2. The localizer phase used a different set of scene pictures, along with scrambled scene pictures, neutral faces, and object pictures. All picture stimuli across all tasks were color photos scaled to the same size (500 × 500 pixels), equalized for overall brightness, and were displayed 7 degrees from the right or 7 degrees from the left of fixation**.**

#### Procedure

Prior to the fMRI session, participants practiced the tasks outside of the MRI scanner. Practice consisted of self-paced reading of written explanations of the fixation, context learning, DNMS, and localizer tasks in addition to a fixed number of practice trials of each task. Participants were encouraged to ask questions in case they needed any instruction clarification. After participants reported that they understood the instructions, they completed another practice trial of the context-learning task and DNMS task in the scanner.

After practice in the scanner, participants were given 5 minutes of fixation training, during which pictures appeared 7 degrees from the right or left of fixation. The goal of this training was to ensure participants perceived the context pictures as lateralized, rather than turning their gaze directly to the picture. We used an EyeLink 1000 eye tracker (SR Research, Ontario, Canada) to give participants real-time feedback; if participants looked away from fixation, then the images would disappear and an “*X*” would appear in the center of the screen until fixation was reestablished.

After fixation training, participants completed the context-list-learning and DNMS tasks described in Experiment 2. Trials in which participants did not respond before the 4-second deadline were excluded from analyses, since there was no response time for these trials.

In the final localizer phase, participants performed a localizer task that was used to discriminate regions of the cortex that preferentially process left and right lateralized face and scene pictures. In this task, pictures were presented one at a time, and participants were asked to press a key indicating whether the currently presented picture was the same as the one immediately preceding. Pictures were presented in miniblocks of 10 presentations each. Eight of the images in each block were trial unique, and two were repeats. Stimuli in each miniblock were chosen from a large stimulus set of pictures not used in the main experiment, and each belonged to one of four categories—faces, objects, scenes, or phase-scrambled scenes—and were presented on either the left or right side of the screen. Thus, there were eight different kinds of miniblock: left face, right face, left object, right object, left scene, right scene, left scrambled scene, and right scrambled scene. Pictures were each presented for 500 ms, and followed by a 1.5-second intertrial interval. Participants completed a total of 24 miniblocks (three blocks per four picture categories presented on either side of the screen), with each miniblock separated by a 12-second interblock interval.

**Fig. 5:**
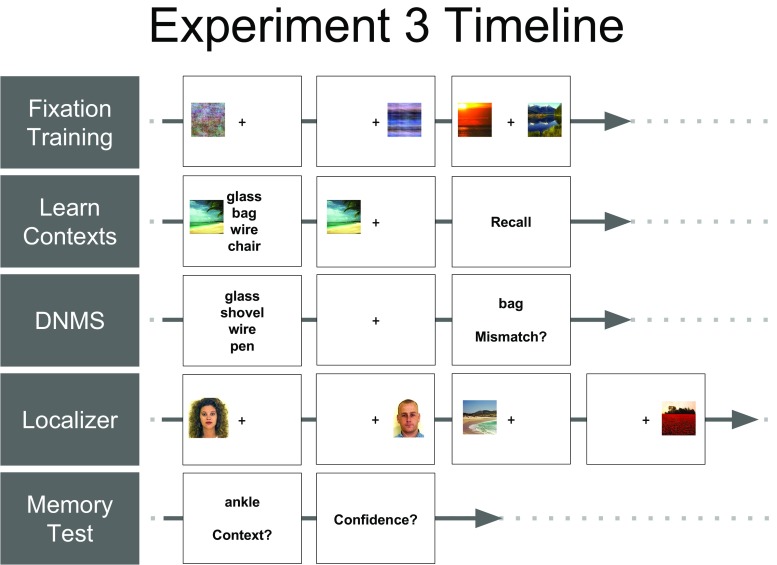
Experiment 3 timeline. We first trained participants to fixate on the center of the screen to ensure that they correctly encoded pictures as being presented on the left or right sides of space. Next, participants associated each of four “contexts” (two pictures of faces and two pictures of scenes) with a unique set of 12 words. The order in which faces/scenes were displayed on the left/right was randomized across participants. Participants then performed the DNMS task from Experiment 2, after which they performed a one-back localizer task involving blocks of face, scene, object, and scrambled scene images presented on the left/right. Images used during the localizer were distinct from the task stimuli. Finally, participants reported the context with which they thought each word was associated during the initial context-learning phase

Finally, after the scanned portions of the experiment had completed, participants remained in the scanner to complete a memory task. Participants were shown each of the 48 words from context learning, one at a time, above all four context pictures, and asked to report both which context was correct and their confidence about that judgement, between one (low confidence) and four (high confidence). A complete timeline of the experiment can be seen in Fig. 5.

#### Imaging methods

##### Data acquisition

Functional magnetic resonance images (fMRI) were acquired during Phases 2, 3, and 4: context learning, DNMS test, and localizer. Data were acquired using a 3T Siemens Prisma scanner (Siemens, Erlangen, Germany) with a 64-channel volume head coil, located at the Princeton Neuroscience Institute. Stimuli were presented using a rear-projection system (Psychology Software Tools, Sharpsburg, PA). Vocal responses were recorded using a fiber optic noise cancelling microphone (Optoacoustics, Mazor, Israel), and manual responses were recorded using a fiber-optic button box (Current Designs, Philadelphia, PA). A computer running MATLAB (Version 2012b, MathWorks, Natick, MA) controlled stimulus presentation.

Functional brain images were collected using a T2*-weighted gradient-echo echo-planar (EPI) sequence (44 oblique axial slices, 2.5 × 2.5 mm inplane, 2.5 mm thickness; echo time 26 ms; TR 1000 ms; flip angle 50°; field of view 192 mm). To register participants to standard space, we collected a high-resolution 3-D T1-weighted MPRAGE sequence (1.0 × 1.0 × 1.0 mm voxels).

##### FMRI data preprocessing

Preprocessing was performed using FSL 5.0.6 (FMRIB’s Software Library, www.fmrib.ox.ac.uk/fsl). The first eight volumes of each run were discarded. All images were skull-stripped to improve registration. Images were aligned to correct for participant motion and then aligned to the MPRAGE. The data were then high-pass filtered with a cutoff period of 128 seconds; 5 mm of smoothing was applied to the data.

##### Region-of-interest definition

Our anatomical regions of interest were fusiform gyrus, parahippocampal gyrus, and lingual gyrus, based on previous reports of visual category-selective patches of cortex—faces (Kanwisher, McDermott, & Chun, 1997) and scenes (Epstein & Kanwisher, 1998). We created a bilateral mask combining these three regions that was used for all pattern classifier analyses. Masks were made using cortical parcellation in FreeSurfer with the Destrieux cortical atlas.

##### Multivariate pattern analysis

We extracted the time series of blood-oxygen-level-dependent (BOLD) signal in our anatomical regions of interest during the localizer task and labeled each TR according to the category miniblock to which it belonged. These labeled time series were used to train an L2-regularized multinomial logistic regression classifier (Polyn, Natu, Cohen, & Norman, 2005) to predict the four class labels (left face/right face/left scene/right scene). In our classifier, the probabilities that each class is present do not sum to 1 because we do not assume the categories are mutually exclusive (e.g., we do not assume that the presence of left face evidence necessarily indicates right face absence; Lewis-Peacock & Norman, 2014). To establish the sensitivity of our classifier to the four categories of interest, we performed a leave-one-out cross-validation. First, we split the MRI data from the localizer phase into four runs by time. Then, we trained the classifier on three of the runs, and tested its performance on the fourth, repeating this procedure once using each run as the holdout set. The resulting average performance was significantly above chance (chance = 25.00%, mean classifier accuracy = 66.99%, *SD* = 18.30%), *t*(35) = 14.1419, *p* < .001, one-sample *t* test compared to chance).

To examine how context reinstatements during the DNMS task affected RTs, we divided DNMS trials into three time periods: the period when the target words were presented (target presentation), the delay period during which participants only saw a fixation cross (delay period), and the period during which participants saw the probe word and had to respond (probe presentation). To account for the hemodynamic lag, we first shifted our TRs by 5 seconds. Our TRs of interest for each event included TRs from 0 to 6 seconds after each event onset (target presentation, delay period start, probe presentation) plus the shift for hemodynamic lag, with a 1 TR offset between each event in order to minimize contamination of signal between the different periods of interest. The trained classifier was then applied to each volume of activity during these three periods of each trial of the DNMS task. The classifier provided a readout of the probability that the BOLD signal during that volume corresponded to a left-face, right-face, left-scene, or right-scene image; we will refer to this as “left/right face/scene evidence”.

### Experiment 3 results

#### Behavioral results

**Fig. 6:**
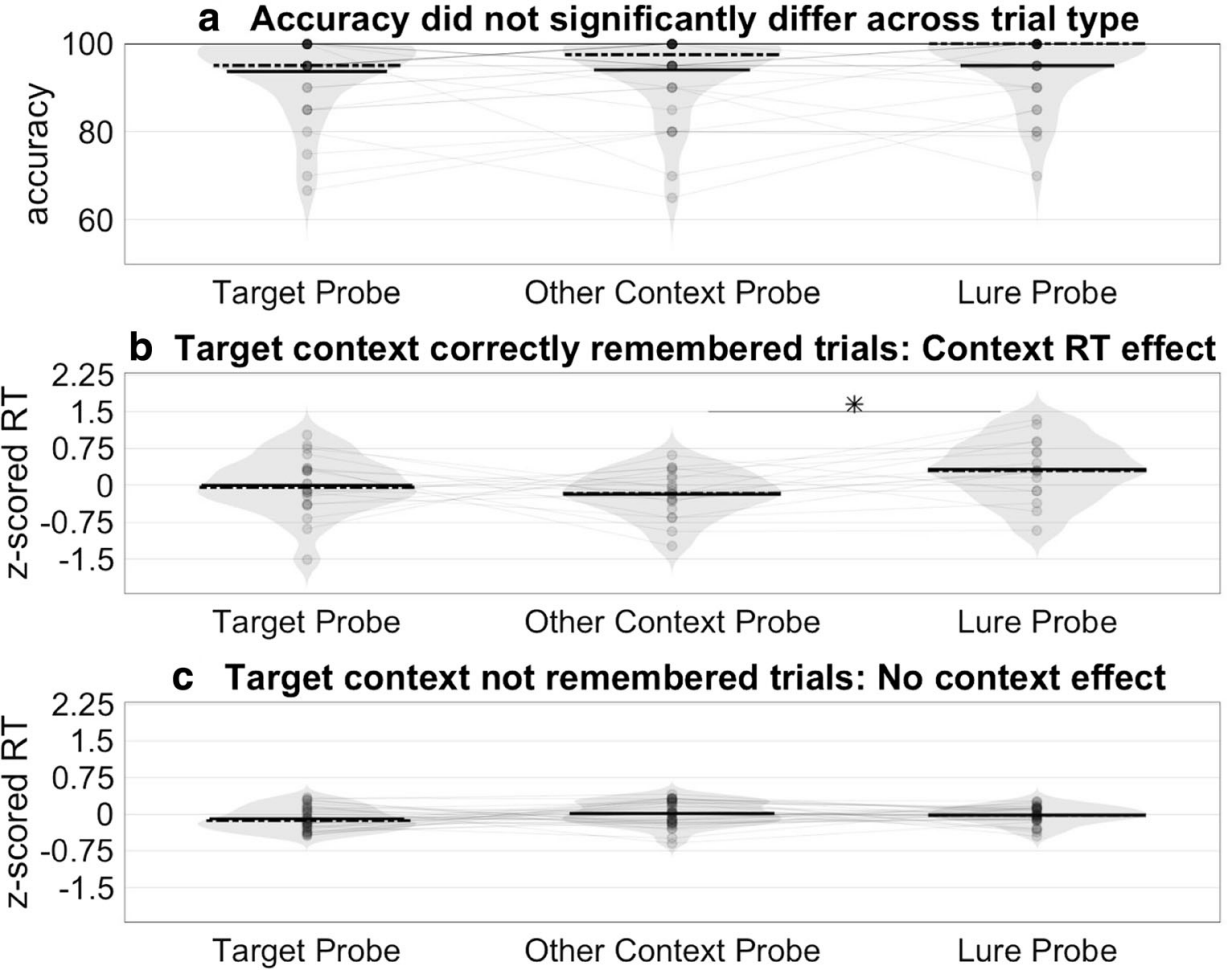
Experiment 3 behavioral results: RT slowdown only seen on lure probe trials when subjects learned the target context. **a** Accuracy did not differ across the three trial types. Solid horizontal black lines reflect mean values; dashed horizontal black lines reflect median values. **b** For trials in which subjects learned to pair the correct context with the target words, subjects were slower to respond to lure probes compared with other-context probes. RTs were log-transformed and *z*-scored within subject to control for individual differences in mean RTs and nonnormal RT distributions. *p < .05. **c** For trials in which subjects did not correctly pair the target words with the target context, there was no difference in RTs across the three conditions

Accuracy for all reported participants was above chance: mean accuracy = 87.27%, *SEM* = 2.97%. Overall, accuracy on Experiment 3 was significantly lower than mean accuracy on Experiment 2 (unpaired two-sample *t* test), *t*(114) = 3.3797, *p* < .001. As in Experiment 2, accuracy did not differ between the three trial types (target: mean = 84.44%, *SEM* = 3.73%; other context: mean = 86.25%, *SEM* = 3.82%; lure: mean = 87.22%, *SEM* = 3.76%; paired, two-sided *t* tests, all *p*s > .2; see Fig. 6a).

Due to time restrictions, three participants were not able to complete the posttask word/context memory test. The 33 participants who completed the test performed above chance, as a group (chance = 25%, mean accuracy = 41.20%, *SEM* = 3.33%), *t*(32) = 4.8648, *p* < .001, two-sided, one-sample compared-to-chance *t* test), and for 25/33 participants individually (proportion *p* < .001 by binomial test).

In contrast to Experiment 2, there was no difference between average RTs in the two mismatch probe conditions (other-context mean log-transformed, *z*-scored RT = .0311, *SD* = .2313; lure mean = .0321, *SD* =.1780), *t*(35) = −.0178, *p* = .9859, paired-sample, two-sided *t* test. However, separating trials where subjects correctly identified which context the target words came from—versus trials where they did not correctly identify the target context words—revealed that the context-based slowdown only occurred when subjects remembered the target context. This was true for both log-transformed *z*-scored RTs (see Fig. 6b) and raw RTs (see Supplemental Fig. S2). Transformed RTs were slower on lure trials than on other-context probe trials (*p* = .03, paired *t* test; see Fig. 6b).

Given the lower overall accuracy on the DNMS task compared with Experiment 2 and the low average word/context memory test scores, it is possible that Experiment 3 participants did not learn the contexts as well as the Experiment 2 subjects; we hypothesized that participants would only show the context related RT effect if they successfully learned the contexts.

Results from a linear mixed-effects regression model that included all trials also supported the hypothesis that the slowing on lure trials in Experiment 2 was driven by reinstated context; the more that target words were correctly identified in the context memory test in Experiment 3, the slower the RTs were for lure trials (β = 11.42, 95% CI [1.55, 21.28], *p* = .02; see Model 1). In this analysis, we estimated the effect of correctly identifying the context belonging to the target words on RTs for each trial type using a mixed-effects linear-regression model. Remembering the context associated with the target words did not significantly affect RTs on target or other probe trials, suggesting the slowdown effect of context was selective to trials where context information was misleading (i.e., lure trials).

**Table:** 

RT ~ 1 + TargetMemoryScore × TrialType + (1 | Subject) **Model 1** We examine the fixed effects of the different trials (TrialType) and correctly remembering the context belonging to the target word (TargetMemoryScore) on reaction time (RT). We also examine the interaction between the two factors to see whether remembering the target words’ context affects RTs differently on the different trial types. We control for idiosyncratic individual subject differences by including (1|Subject). All trial types were included in this analysis. Inaccurate trials were excluded from analysis.	

#### FMRI results

We trained an fMRI pattern classifier to discriminate between the four encoding contexts. Then we measured evidence that subjects were reinstating, we measured evidence that subjects were reinstating the encoding context associated with the target and probe words. Our classifier did not assume that subjects could only think about one context at a time (e.g., the classifier could find simultaneous evidence for faces on the left and right; Lewis-Peacock & Norman, 2014).

We tested whether participants were more likely to reinstate the context associated with the target words than the other contexts. For each subject, we computed the average amount of target context minus nontarget context evidence and compared this value against zero. Over all subjects, there was significantly more target context evidence than nontarget evidence, *t*(35) = 3.34, *p* = .002, one-sample *t* test.

We predicted that, on lure trials, greater reinstatement of the context associated with the target and probe word would cause subjects to be slower to respond, on the assumption that greater activity of the probe word in working memory will make it harder to identify the probe as a mismatch. On target trials, in which the probe word actually was one of the targets, we predicted that reinstating the probe-word context would not slow performance.

First, we tested whether context reinstatement led to slowed responses. We estimated the effect size of probe-context reinstatements during our time periods of interest using a mixed-effects linear-regression model for each trial type (see Model 2). Supporting our hypothesis, greater evidence for delay-period reinstatement of the probe context was significantly associated with slowed responses on lure trials (β = 34.62, 95% CI [9.34, 59.89], *p* = .007).

**Table:** 

RT ~ 1 + ProbeContextReinstatementTargetsPresentation + ProbeContextReinstatementsDelay + ProbeContextReinstatementsProbePresentation + (1 | Subject) **Model 2** We examine the fixed effects of reinstating the probe-word’s context during different periods of the DNMS trial on reaction time (RT). ProbeContextReinstatementTargetsPresentation refers to reinstatements of the probe-word’s context during presentation of the targets. The same naming convention applies to probe-context reinstatements during the delay period (ProbeContextReinstatementsDelay) as well as during the probe presentation period (ProbeContextReinstatementsProbePresentation). We control for idiosyncratic individual subject differences by including (1|Subject). This model was run separately for each trial type. Inaccurate trials were excluded from analysis.	

**Fig. 7:**
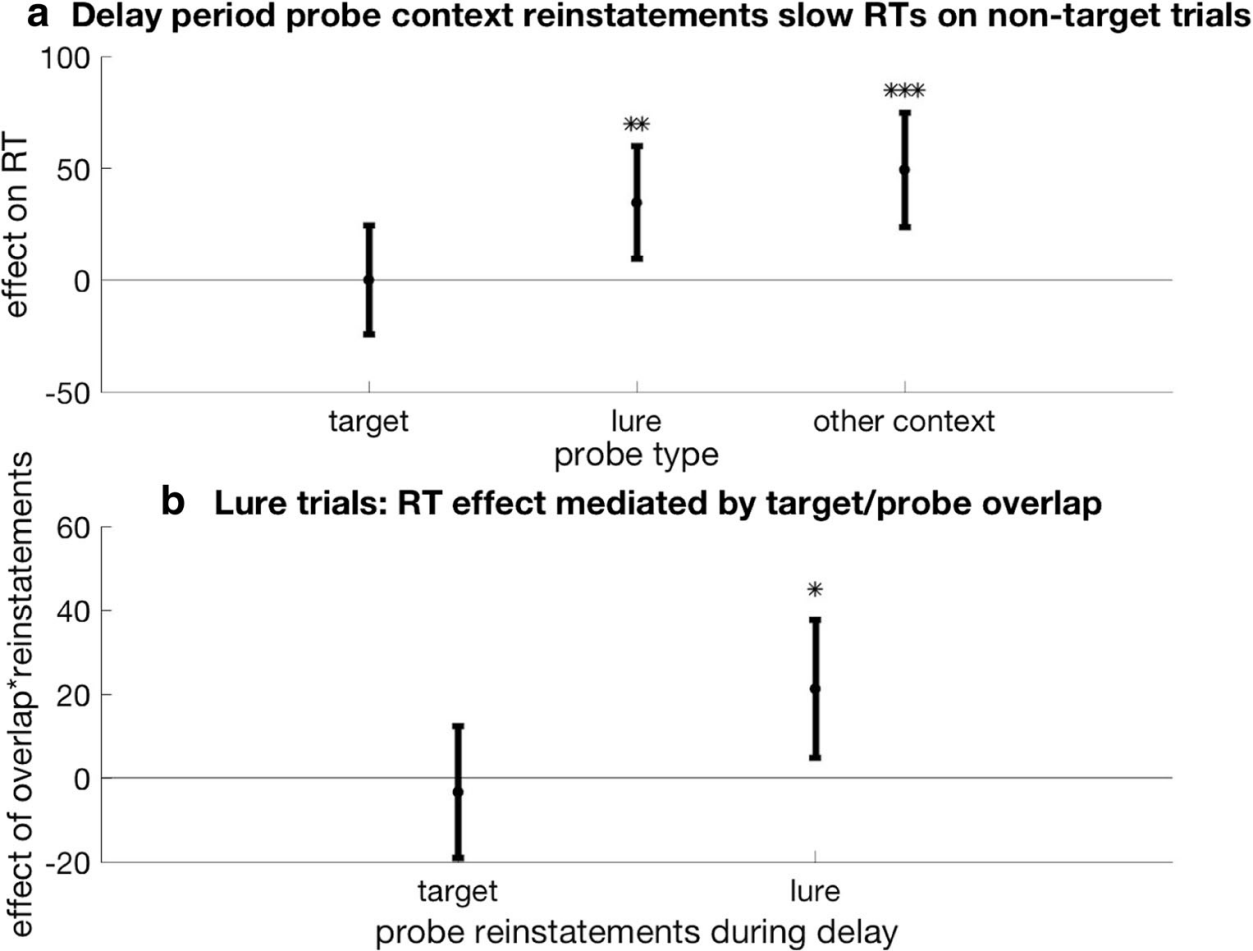
**a** Greater evidence for delay-period reinstatement of the probe context was associated with slower RTs on lure trials (β = 34.62, 95% CI [9.34, 59.89], *p* =.007) and other-context probe trials (β = 49.37, 95% CI [23.72, 75.02], *p* < .001). Reinstating the probe context during the delay period on target trials did not slow RTs, potentially because these reinstatements did not introduce misleading information into WM on these trials (β = 0.03, 95% CI [−24.30, 24.36], *p* = .99). **b** For lure trials, we predicted that context reinstatements during the delay period would be more likely to slow RTs if the lure was directly associated not just with the context picture but also with the target words. The more often the probe and targets were encountered together during context learning, the more likely participants were to exhibit a slowed RT after reinstating the misleading probe context on lure trials (β = 21.28, 95% CI [4.85, 37.71], *p* = .01). This analysis was limited to lure trials because other-context probes never overlapped with the targets. **p < .01., ***p < .001. Vertical bars reflect 95% CI. Inaccurate trials were excluded from analyses in Fig. 7a–b to minimize the effect of attentional lapses on RT results. (See Supplemental Fig. S3 for analyses including all trials)

Following the same logic as the lure trials, we found that reinstating the probe context on other-context probe trials (and thus potentially introducing the other-context probe into working memory) also slowed RTs (β = 49.37, 95% CI [23.72, 75.02], *p* < .001; see Fig. 7a). (Probe context reinstatements were also observed to slow RTs on lure and other-context trials when all trials were included in the model, with trial type included as an interaction term: β = 38.74, 95% CI [8.25, 69.23], *p* = .01.)

Reinstating the probe context during the delay period on target trials did not slow RTs (β = 0.03, 95% CI [−24.30, 24.36], *p* = .99; see Fig. 7a), possibly because these reinstatements did not introduce misleading information into working memory (as these words were just presented and thus should already be in working memory).

On lure and other context probe trials, we found reinstating the nonprobe context during the delay period actually speeded responses (lure trials β = −96.10, 95% CI [−171.25, −20.95], *p* = .01; other-context trials β = −137.16, 95% CI [−213.49, −60.844, *p* < .001; Model 2 run with nonprobe context reinstatements instead of probe context reinstatements). Thus, it does not appear that all context reinstatements have the same effect on behavior; misleading (probe context) reinstatements significantly slowed RTs while nonmisleading (nonprobe context) significantly sped reinstatements.

We hypothesized that the context-based RT effect seen on lure trials would be further mediated by the degree of association between the target words and lures. To test this, we exploited a feature of our experiment that allows us to dissociate between the effects of reinstating pictures versus words: While each word was seen the same number of times with its context picture, there was variation in the number of times each word was presented with another word from the same context during context learning. For each DNMS trial, we computed the number of times the targets and probe were presented together during encoding, a number we called “overlap”; across subjects and trials, overlap scores ranged from 0 to 7 (mean = 3.62, *SD* = 1.50).

We predicted that context reinstatements during the delay period would be more likely to slow RTs if the probe word was directly associated not just with the context picture but also with the target words (i.e., had higher overlap scores). We used a linear mixed-effects regression model to examine how the overlap between the probe and the targets interacted with probe context reinstatements to predict RTs. This analysis was restricted to target and lure trials only, as, by definition, probes on other-context trials were never presented with the target words (see Model 3).

**Table:** 

RT ~ 1 + ProbeContextReinstatementDelay × Overlap + (1 | Subject) **Model 3** We examine the interaction between the number of times the probe word and target words were presented together (Overlap) and the effect of reinstating the probe-word’s context on reaction time (RT). ProbeContextReinstatementsProbePresentation refers to reinstatements of the probe-word’s context during the delay. We control for idiosyncratic individual subject differences by including (1|Subject). Only lure-probe trials were included in this analysis. Inaccurate trials were excluded from analysis.	

We found significant interaction between overlap scores and evidence for probe-context reinstatement on lure trials (β = 21.28, 95% CI [4.85, 37.71], *p* = .01; see Fig. 7b): The more often a given probe overlapped with target words, the more effective reinstatements were at slowing reaction times on lure trials. There was no effect of overlap on RTs for target trials (β = −3.49, 95% CI [−19.15, 12.18], *p* = .66).

### Experiment 3 discussion

Experiment 3 revealed that memories reinstated during the delay period can alter the contents of working memory, even when these intrusions negatively impact performance on an upcoming match to sample probe.

Using fMRI, we showed that this effect is specific to the degree, timing, and episodic content of the reinstated memories. Namely, disruption results only from context information reinstated during the maintenance period, as opposed to during target or probe presentation. Further, underscoring the episodic nature of these intruding memories, the effect was greater when the potentially misleading words had been presented alongside the target words.

Taken together, these results demonstrate that ongoing episodic memory reinstatement intrudes on working-memory maintenance.

## General discussion

By maintaining a high-fidelity record of recent information, working memory allows us to perform tasks that require accurate storage over short periods of time. However, the presence of distraction or the need to focus on a new task can compromise that record and impair performance. Episodic memory complements these characteristics by storing memories over a longer term, at the cost of reduced fidelity and the risk of retrieval failure (Cohen & O’Reilly, 1996; McClelland et al., 1995; O’Reilly & Rudy, 2001).

While the identification and study of these distinct systems has benefited from efforts to isolate them, it seems unlikely that they would operate entirely independently of one another under natural conditions. Regions that exhibit activity associated with the performance of episodic memory tasks have been observed to be active even during rest, suggesting ongoing replay of episodic memories (Carr, Jadhav, & Frank, 2011; Jadhav, Kemere, German, & Frank, 2012; Wilson & McNaughton, 1994). These memory reinstatements can lead to the incidental reinstatement of the context in which the memories were experienced (Bornstein & Norman, 2017). These reinstatements have also been observed to involve coordinated activity across the entire brain, including prefrontal areas associated with working-memory maintenance (Miller & Cohen, 2001). Thus, in a manner analogous to externally driven stimuli, internally driven reinstatements from episodic memory may also impact representations stored in working memory.

Over a series of three experiments, we tested the hypothesis that episodic memory reinstatement influences performance under task conditions traditionally used to assess working memory maintenance, even in the absence of external interference. In Experiment 1, we showed that, when working memory maintenance is disrupted in a delayed-recall task, participants intrude other items from the same context as the studied target items.

Experiment 2 revealed that, even when accuracy is near ceiling, other measures of performance can detect intrusions from episodic memory. On a delayed nonmatch-to-sample task (DNMS) with a distraction-free 18-second delay, participants were slowed in their responding to lure probes—words that shared an encoding context with the target set, but which were not actually members of the target set.

Experiment 3 repeated the DNMS task from Experiment 2. Consistent with the possibility that task-irrelevant context information can affect behavior, we found that participants slowed down on lure trials when they had correctly encoded the context belonging to the target words. Using fMRI in Experiment 3 allowed us to investigate the behavioral effects of episodic memory when it was engaged. This analysis revealed that the specific content of episodic-memory reinstatement during the delay period predicted the degree of response slowing on that trial.

### The function of reinstatements during working-memory maintenance

We have provided evidence that reinstatement of recent experiences from episodic memory has specific, measurable influence on the contents of working memory, even over short delay periods in the absence of explicit interference. Why is working memory influenced by episodic-memory reinstatement, even under these conditions? The effect of episodic-memory contents on working memory could simply be a side effect, or it could indicate that laboratory tests of working-memory maintenance obscure key features of the way that working memory operates in more naturalistic environments. One possibility is that episodic memory is recruited by control mechanisms to “refresh” decaying or disrupted representations.

While some of these reinstatements may be strategically directed recalls in service of maintaining decaying working-memory representations, others may instead be ongoing reinstatements of the sort associated with resting-state activity or forward planning (Deuker et al., 2013; Foster & Wilson, 2006; Tambini et al., 2010). On this view, the ability to interact with working memory may be an adaptive feature of resting-state reinstatements from episodic memory—in other words, it may not just sustain but also transform working-memory representations, by integrating information in working memory with information from recent events. That these reinstatements include contextually related events implies that such an interaction could support rapid, goal-relevant generalizations (Collins & Frank, 2012; Kumaran & McClelland, 2012; Kumaran, Summerfield, Hassabis, & Maguire, 2009). The mechanism outlined here both constrains, and expands, that proposal, with potentially broad impacts for the study of memory-guided decision-making.

#### Data and code availability

The fMRI data that support the findings of this study are publicly available on OpenNeuro (https://openneuro.org/datasets/ds001576/versions/1.0.0). The behavioral data that support the findings of this study are available on request from the corresponding author. The behavioral data are not yet publicly available because they contain information that could compromise research participant privacy, such as vocal recordings. All software used to analyze the data are free and publicly available. Standard software packages (SPM8 and FSL 5.0.4) were used for preprocessing the MRI data. The Princeton MVPA toolbox (https://github.com/PrincetonUniversity/princeton-mvpa-toolbox) was used to perform MVPA analyses.

## Electronic supplementary material

ESM 1 (DOC 547 kb)
