# Supplementary material for: Refresh my memory: Episodic memory reinstatements intrude on working memory maintenance
Source: Cogn Affect Behav Neurosci. 2018 Dec 4;19(2):338–54. doi: 10.3758/s13415-018-00674-z (PMC6420448; doi:10.3758/s13415-018-00674-z)
Supplement: Supplementary file 1 — (DOC 547 kb) [file 13415_2018_674_MOESM1_ESM.doc]

## **Supplement**


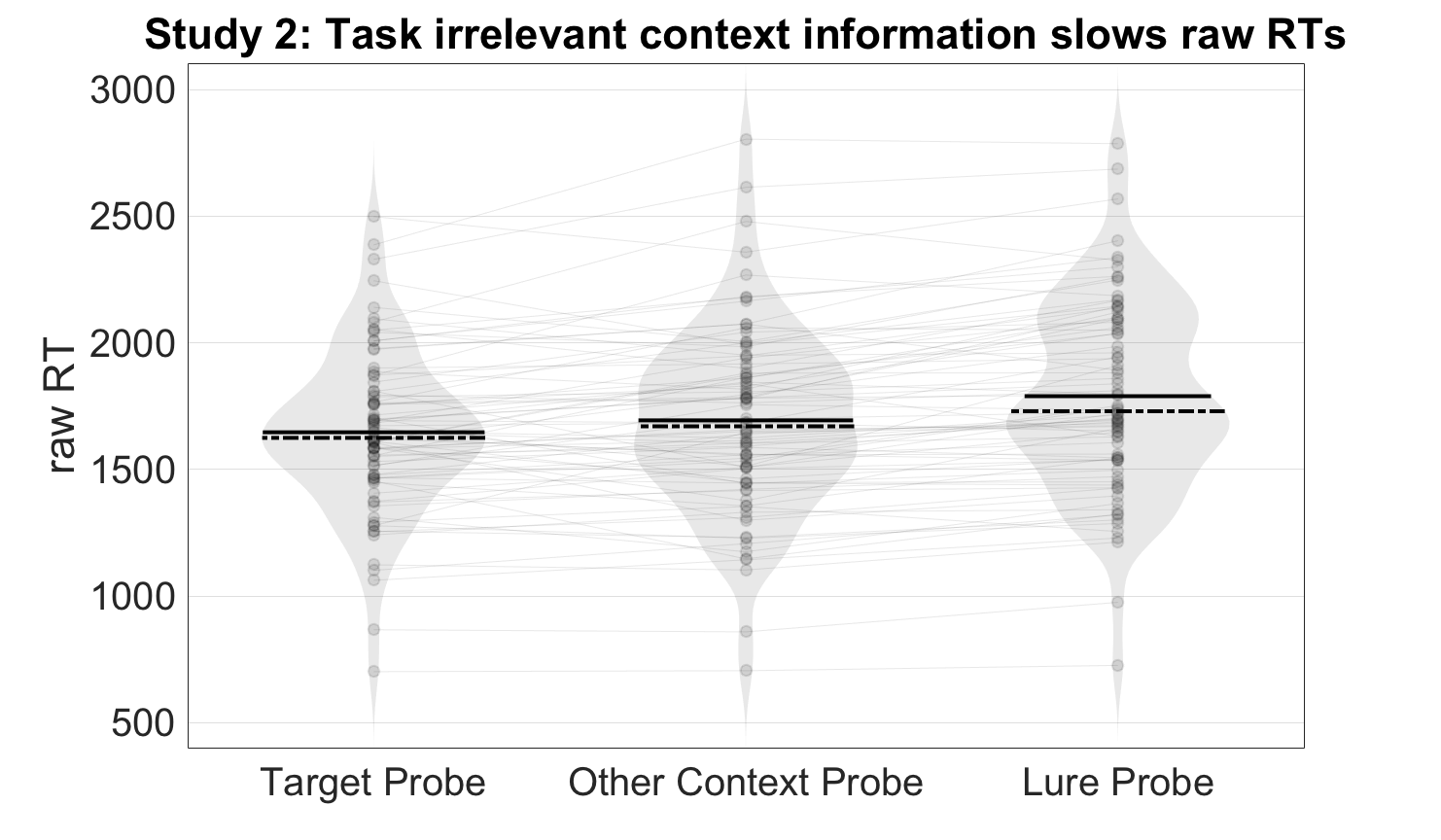


#### **Supplemental Figure 1.** Same analysis as shown in Figure 4B, but using raw RTs. Using paired, two-sided t-tests, we found that participants responded more slowly to *lure probes* (mean RT = 1789.6 ms, std = 381.0 ms), than to *target probes* (mean RT = 1647.2 ms, std = 321.2 ms; *t*(79) = -7.6318, *p* < .001), or *other-context* probes (mean RT = 1694.4 ms, std = 366. 9 ms; *t*(79) = -7.0489, *p* < .001). The latter is noteworthy, as the the only difference between these lure and other-context probes is whether the probe word was learned in the same context as the target during the task-irrelevant part of the experiment.Solid black lines represent mean RT, dashed lines represent median RT. * = *p* < .05, *** = *p* < .001.


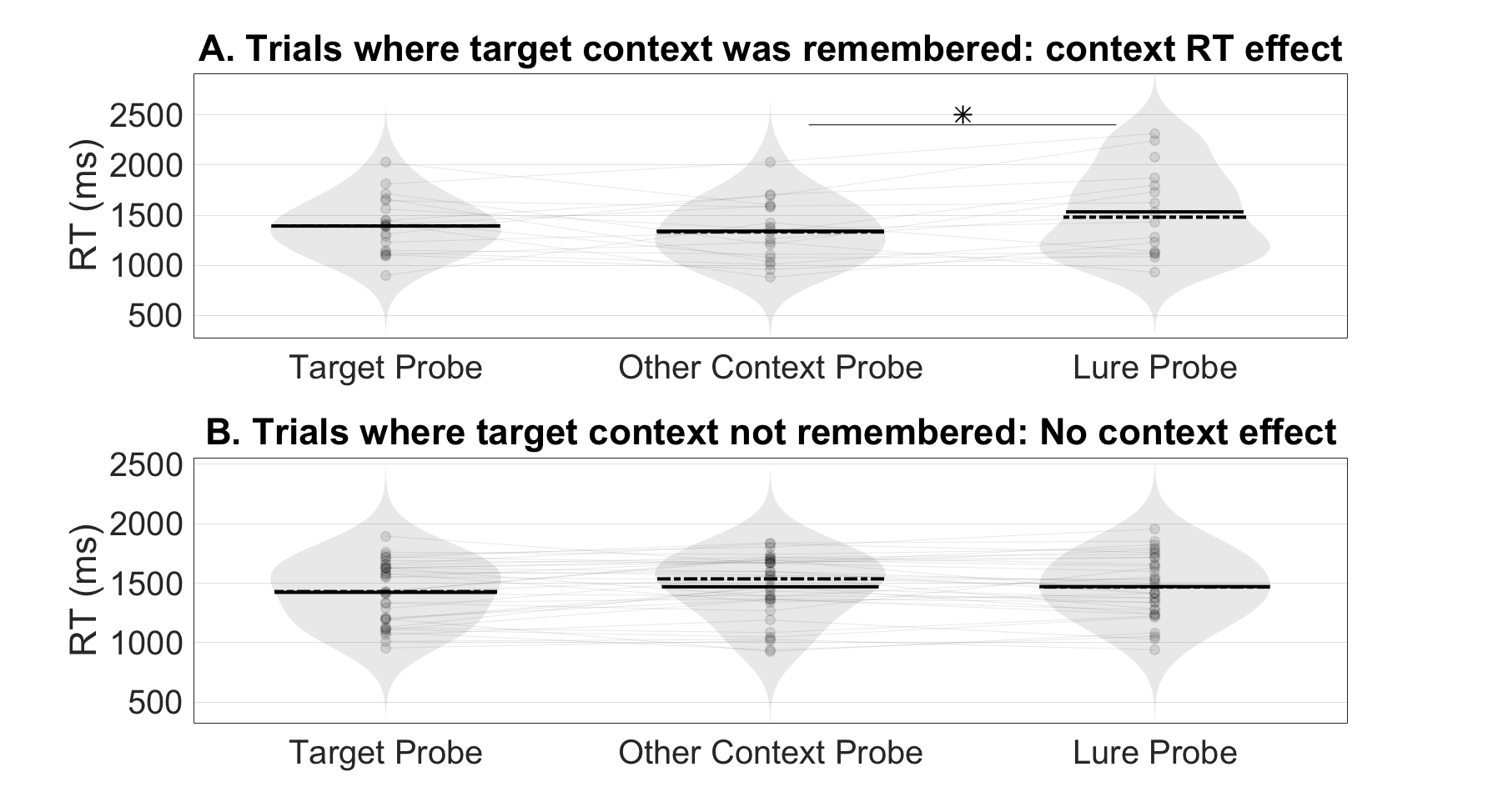


**Supplemental Figure 2. Experiment 3 behavioral results: RT slowdown only seen on lure probe trials when subjects learned the target context.** A. For trials in which subjects learned to pair the correct context with the target words, subjects were slower to respond to lure probes compared to other-context probes.This same pattern was seen in raw RTs as well as transformed RTs (Figure 6B). Solid horizontal lines reflect mean values, dashed horizontal lines reflect median values. * = p < .05. B. For trials in which subjects did not correctly pair the target words with the target context, there was no different in RTs across the three conditions. This same pattern was seen in raw RTs as well as transformed RTs (Figure 6C).


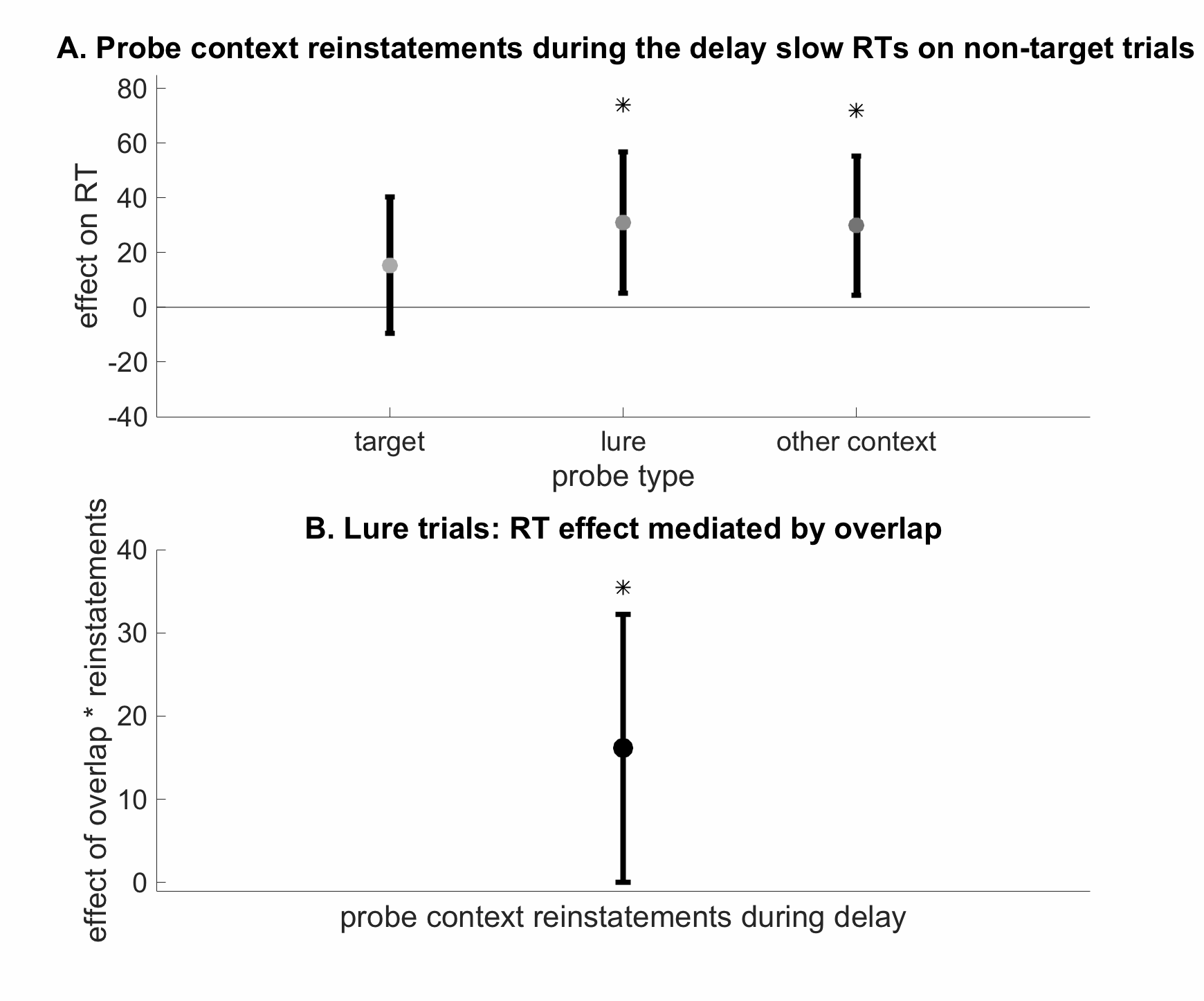


**Supplemental Figure 4. Context-based RT effect evident across all DNMS trials (both accurate and inaccurate). A.** Across both accurate and inaccurate DNMS trials, greater evidence for delay-period reinstatement of the probe context was associated with slowed responses on lure trials (β = 29.87, 95% confidence interval = [4.39 55.3], *p* =.02), as well as other-context probe trials (β = 31.0 95% confidence interval = [5.13 56.87], *p* = .02). Reinstating the probe context during the delay period on target trials did not slow RTs, since these reinstatements did not introduce misleading information into working memory on these trials (β = 15.4, 95% confidence interval = [-9.62 40.42], *p* = .23). *** indicates *p <* .05. Vertical bars reflect 95% CI. **B.** We predicted that context reinstatements during the delay period would be more likely to slow RTs if the probe word was directly associated not just with the context picture, but also with the target words. For each lure trial, we calculated the number of times the probe word and target words were encountered together during context learning. Across both accurate and inaccurate DNMS trials, we found that the more often the probe and targets were encountered together, the more likely participants were to exhibit a slowed response after reinstating the misleading probe context (β = 16.15, 95% confidence interval = [.07 32.22], *p* = .04). Vertical bars reflect 95% CI. * indicates *p* < .05.

| **Model 1** **Factors** | **β** | **95%CI: Lower** | **95%CI: Upper** | **tStat** | ***p*** |
| --- | --- | --- | --- | --- | --- |
| Intercept | 1401.30 | 1309.90 | 1492.70 | 30.06 | <.0001 |
| LureProbe | 13.93 | -53.82 | 81.68 | 0.40 | .69 |
| OtherContextProbe | 52.26 | -15.84 | 120.37 | 1.51 | .13 |
| TargetContextMemoryScore | 2.43 | -5.00 | 9.86 | 0.64 | .52 |
| LureProbe * TargetContextMemoryScore | 11.42 | 1.55 | 21.28 | 2.27 | .02* |
| OtherContextProbe * TargetContextMemoryScore | -0.22 | -9.70 | 9.26 | -0.04 | .96 |

**Supplemental Table 1. Full results from Model 1 analyses.** Linear mixed effects regression Model 1 included all accurate DNMS trials. Lure trials were not overall slower than other-context probe trials. However, the more target words were correctly identified in the context memory test in Experiment 3, the slower the RTs were for lure trials. Remembering the context associated with the target words did not significantly affect RTs on target or other probe trials, suggesting the slow-down effect of context was selective to trials where context information was misleading (i.e. lure trials).

| **Model 2** **Factors: Target Trials** | **β** | **95%CI: Lower** | **95%CI: Upper** | **tStat** | ***p*** |
| --- | --- | --- | --- | --- | --- |
| ProbeContextReinstatements: TargetPresentation | -12.90 | -40.70 | 14.90 | -0.91 | .36 |
| ProbeContextReinstatements: DelayPeriod | 0.03 | -24.30 | 24.36 | 0.00 | .99 |
| ProbeContextReinstatements: ProbePresentation | 3.13 | -18.10 | 24.37 | 0.29 | .77 |
| **Model 2** **Factors: Lure Probe Trials** | **β** | **95%CI: Lower** | **95%CI: Upper** | **tStat** | ***p*** |
| ProbeContextReinstatements: TargetPresentation | -41.63 | -71.49 | -11.78 | -2.74 | .006** |
| ProbeContextReinstatements: DelayPeriod | 34.62 | 9.34 | 59.89 | 2.69 | .007** |
| ProbeContextReinstatements: ProbePresentation | 0.67 | -21.97 | 23.30 | .06 | .95 |
| **Model 2** **Factors: Other-Context Probe Trials** | **β** | **95%CI: Lower** | **95%CI: Upper** | **tStat** | ***p*** |
| ProbeContextReinstatements: TargetPresentation | -25.59 | -54.99 | 3.80 | -1.71 | .09 |
| ProbeContextReinstatements: DelayPeriod | 49.37 | 23.72 | 75.02 | 3.78 | .0002*** |
| ProbeContextReinstatements: ProbePresentation | 0.62 | -20.58 | 21.83 | .06 | .95 |

**Supplemental Table 2. Full results from Model 2 analyses.** Linear mixed effects regression Model 2 was run separately for each DNMS trial type. For lure trials, probe context reinstatements during the target presentation sped up RTs while probe context reinstatements during the delay period slowed RTs. For lure trials, the probe context and target context were the same (lure probes were drawn from the same context as the targets). Target context reinstatements while the target words were visually displayed could reflect better attention to the target words, leading to better encoding of the targets and faster reaction times to probes. Across subjects and trial types (lure trials and other-context trials) reinstating the context associated with a misleading probe during the delay period slowed reaction times.

RT ~ 1 + ProbeContextReinstatements * TrialPeriod + (1 | Subject)

Supplemental Model 2: We examine the interactions between probe context reinstatements (ProbeContextReinstatements) during the different periods of the trial (target presentation, delay period, probe presentation; TrialPeriod), for lure and other context trials. We control for idiosyncratic individual subject differences by including (1|Subject). Inaccurate trials were excluded from analysis.

**Supplemental Model 1. Testing difference in effect sizes for Model 2.** To test whether the effects of interest from Model 2-- probe context reinstatements slowing reaction times during lure and other context probe trials--were significantly different during the delay period than other time periods, we entered lure and other context probe trials into a linear mixed effects model testing the interaction between context reinstatements and period of trial. We coded the maintenance period as the default period to which target period and probe period reinstatements could be compared. We found probe context reinstatements during the delay period affected behavior significantly differently than probe context reinstatements during target presentation (β = -57.57, *p* < .001, 95% CI = [-88.13 -27.01]), as well as during probe presentation (β = -27.23, *p* = .04, 95% CI = [-53.20 -1.26]).
